# Hydrate of Chloral

**Published:** 1871-04

**Authors:** H. N. Hurlbut

**Affiliations:** Chicago


### HYDRATE OF CHLORAL.

CHICAGO, *February 10, 1871.*

DR. N. S. DAVIS,—SIR,—In noting your article on a "Large Dose of Hydrate of Chloral," and your suggestions in regard to the difference of quality of the chloral in our drug stores, and the great variations in the capacity of different patients to sustain life under its effects, induced me to make this suggestion, that it depends more on the alkaline state of the patient's system at the time of the administration, or the administration of an alkali soon after the chloral was taken; for, in either case, the chloroform will be more rapidly liberated from the chloral, and its effect more rapid and powerful.

Such has been my observation. Your situation in Mercy Hospital will enable you to confirm or correct my suggestions.

If correct, would it not go far in accounting for many of the unpleasant cases recorded against hydrate of chloral.

If your investigations should prove that alkalies have that effect, I think it could not be too widely known.

H. N. HURLBUT, M.D.

NOTE,—Is there not danger of a chemical change taking place where chloral is held in solution a few days? Do you find as marked an effect from a given dose on the third as on the first day of administration? I do not.

